# The use of capture-recapture methods to provide better estimates of the burden of norovirus outbreaks from seafood in England, 2004–2011

**DOI:** 10.1017/S0950268818003217

**Published:** 2018-12-04

**Authors:** J.L. Hardstaff, H.E. Clough, J.P. Harris, J.A. Lowther, D.N. Lees, S.J. O'Brien

**Affiliations:** 1 Farr Institute @ HeRC, Block F –floor 2, Waterhouse Building, University of Liverpool, Liverpool, L69 3GL, UK; 2 NIHR Health Protection Research Unit in Gastrointestinal Infections, Liverpool, UK; 3 Centre for Environment Fisheries and Aquaculture Science, Barrack Road, Weymouth, Dorset, DT4 8UB, UK

**Keywords:** Capture-recapture analysis, food safety, gastrointestinal infections, norovirus, outbreaks

## Abstract

Norovirus (NoV) is the greatest cause of infectious intestinal disease in the UK. The burden associated with foodborne outbreaks is underestimated in part because data are dispersed across different organisations. Each looks at outbreaks through a different lens. To estimate the burden of NoV from seafood including shellfish we used a capture-recapture technique using datasets from three different organisations currently involved in collecting information on outbreaks. The number of outbreaks of NoV related to seafood including shellfish in England was estimated for the period of 2004–2011. The combined estimates were more than three times as high (*N* = 360 using Chao's sample coverage approach) as the individual count from organisation three (*N* = 115), which captured more outbreaks than the other two organisations. The estimates were calculated for both independence and dependence between the datasets. There was evidence of under-reporting of NoV outbreaks and inconsistency of reporting between organisations, which means that, currently, more than one data source needs to be used to estimate as accurately as possible the total number of NoV outbreaks and associated cases. Furthermore, either the integration of reporting mechanisms or simplifying the process of reporting outbreaks to organisations is essential for understanding and, hence, controlling disease burden.

## Introduction

Norovirus (NoV) infection is the commonest cause of diarrhoea and vomiting worldwide [1]. The virus has a low infectious dose and frequently leads to outbreaks [2]. Most infections are acquired through person to person contact, leading to secondary and tertiary transmission [3, 4]. The reservoir for human NoV is the gastrointestinal tract of humans. Consumption of bivalve shellfish, most often raw oysters, is commonly associated with NoV infections [5]. This is usually as a result of contamination of the water in which the oysters are grown and harvested [6]. The virus adheres to the intestinal tract of the oysters and depuration is not effective at removing NoV [7]. In addition, contamination of shellfish by infectious food handlers, as is commonly reported for other food commodities [8], may contribute to the burden of shellfish-related outbreaks.

In the UK, data on outbreaks of NoV associated with shellfish can be reported to three agencies. The mechanisms driving the reporting to each of the three organisations differ. One organisation is responsible for collating data on human illness separating NoV outbreaks in hospitals from those associated with food. A second has food safety as its primary remit and the third takes responsibility for microbiological testing of shellfish. The reporting process takes different pathways depending upon the origin of reports of illness and whether the illness is thought to be foodborne. It is complicated by the fact that symptomatic individuals are discouraged from visiting their general practitioner (GP) to prevent person to person spread of NoV and this also contributes to underreporting [9].

Capture-recapture analyses have been used in epidemiology since Wittes and Sidel's work on estimating the frequency of adverse reactions to methicillin using information from several different sources [10]. They have been used to estimate the number of human foodborne infections with agents such as *Salmonella* [11] and to evaluate the number of cases missing from surveillance programs, for example, scrapie in the UK [12]. Many of the earlier methods which apply capture-recapture methodology in the context of epidemiology for assessing under-ascertainment assume independence of reporting sources. For example, the well-established method of Peterson [13] provides a simple way in which two independent lists can be used together to estimate the true number of individuals in the community. Chapman [14] provides a modified version of the Peterson estimator, which again assumes independence of lists but corrects for biases introduced by a small overlap between lists. If there is positive dependence between the two lists (so that membership of one list makes membership of another more likely), both the Peterson and the Chapman estimators will prove unreliable.

The first objective of the study was to estimate the number of NoV outbreaks associated with seafood in England between the years 2004 and 2011 using outbreak reporting data from three different organisations in England. Capture-recapture methods will be used to estimate the number of outbreaks by observing which of the three organisations receive reports of individual outbreaks and exploiting information on their occurrence with the other two organisations. The second objective was to determine the levels of dependence between pairs of lists and to ascertain whether an outbreak was more or less likely to appear on one list if it had appeared on another to improve the estimate of the number of outbreaks.

## Methods

### Data

Datasets from three different governmental organisations consisted of: date of outbreak; date of reporting; location of outbreaks; type of location e.g. restaurant; the affiliation of the outbreak reporter e.g. local government, member of the public etc.; and details about the outbreak including the pathogen (and strain), suspected food vehicle, number of cases and number at risk where known. Outbreaks were compiled into lists, each list corresponding with one of the three organisations.

An outbreak was defined as cases of NoV reported to an organisation. Organisation one (O1) recorded outbreaks from 2004 and 2015, organisation two (O2) recorded outbreaks from 2003 to 2011 and organisation three (O3) recorded outbreaks from 1995 to 2015. The only common food vehicle for outbreaks for all three organisations was seafood.

### Criteria for inclusion

Outbreaks which originated from England between January 2004 and December 2011 and were associated with seafood were included so that all datasets were temporally comparable. Outbreaks were excluded if they were recorded as occurring in the same establishment (based on the name of the establishment and geographical location) within the same month or the duration of a month that overlapped 2 months within a dataset to avoid counting an outbreak from recontamination by the same source twice. When this occurred the first notification was included in the study and subsequent notification was excluded.

### Matching between organisations

A definite match between sources was determined by date (outbreaks occurring within 2 weeks of each other were deemed to be the same outbreak), the name of the establishment in which the outbreak was recorded and its geographical location.

In cases where matching was performed, information was judged to contain sufficient detail to inspire confidence that the matching was accurate (e.g. using features such as establishment, location and date in combination). Cases, where an outbreak occurred in a private setting, could potentially have been ambiguous but were found to have occurred in different months which enabled a distinction between them to be drawn.

### Statistical methods

#### Estimating the number of outbreaks assuming dependence

Our working hypothesis is that there will be positive dependence between lists given that the same individuals, for example, Environmental Health Officers, may, in practice, be notifying different bodies, either at the same time or within a narrow time-window. To account for this, we initially use the sample coverage approach proposed by Chao and Tsay [15] and illustrated further in Chao *et al*. [16]. We use R package CARE1 [17], run in R version 3.4.1 [18], to calculate Petersen, Chapman and Chao and Tsay population estimates and their associated 95% confidence intervals. Within this, dependence is modelled using a group of parameters called the coefficients of variation. For a pair of lists *j* and *k*, the coefficient of variation between the lists is defined as
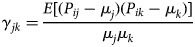
where *P*_*ij*_ is the probability of outbreak *i* being in list *j* and *μ*_*j*_ is the probability being in list *j*, averaged over all outbreaks.

The coefficient of variation relates to the covariance of samples *j* and *k*. The coefficient is generalisable to sets of three lists. The magnitude of *γ*_*jk*_ measures the degree of dependence between samples j and k, with heterogeneous samples being independent if and only if *γ*_*jk*_ = 0. Positive dependence is indicated by *γ*_*jk*_>0 (and similarly negative dependence by *γ*_*jk*_ < 0).

Before estimating the population size *N*, we must describe the overlap between the different groups and combine this information with the information on dependence provided by the *γ*_*jk*_ terms using a quantity called the sample coverage, which was originally proposed by Turing and Good [19] and is expanded upon in the context of under-ascertainment by Chao *et al.* [16]. Consider the three list case. Given an outbreak from list three, to determine overlap, we are interested in the probability that outbreak had also been identified by the combined lists one and two. The overlap fraction is then the sum of the conditional probabilities of each outbreak *i* appearing on list three who also appear on list one and two combined, divided by the sum of the conditional probabilities of all outbreaks *i* appearing on list three (i.e. putting all those who appear on lists one and two combined and those who do not, together). This relationship can be considered for any of the three lists and so the sample coverage is defined as the average of the three possible overlap fractions (list three given list one and two combined; list two given list one and three combined; and list one given list two and three combined). This information is used to produce three estimates of N, of which two are potentially of interest for our purposes: 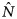, which models dependence between lists and is appropriate if sample coverage proves to be high; and 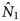, which again models dependence between lists but is appropriate if the estimated sample coverage proves to be low. Confidence intervals (CI) are placed around the population size estimates using bootstrap methods as described in Chao *et al*. [16].

A second approach to accounting for the dependence in the occurrence of outbreaks upon the lists from the different organisations involves the use of Poisson log-linear models as described by Cormack [20]. Models were fitted to the counts of outbreaks occurring on different combinations of lists. Observed counts for each organisation were modelled as Poisson random variables with expectation equal to the true underlying rate of outbreaks multiplied in each case by some function of the outbreak capture process for that organisation. The model assumes that there is a closed population of outbreaks so that the true number of outbreaks is fixed across the duration of the study. The methods derive from ecology where this assumption may be questionable but given that there is no equivalent of birth and death in epidemiological modelling of this nature this assumption is reasonable. Again, capture histories for the organisations are obtained by linking outbreaks. We fit the model including all main effects (O1, O2 and O3) and considering all possible different combinations of lists for interactions ([1,2] and [2,3]; [1,3] and [2,3]; [1,2] and [1,3]; [1,2] only; [1,3] only; [2,3] only): note it is not possible with three lists to include all pairwise combinations as this results in over-parameterisation.

Models were fitted using the Rcapture R package [21] described in Baillargeon and Rivest (2007) [22] in R version 3.4.1 [18]. We compare models using the Akaike Information Criterion (AIC) [23].

#### Exploring sources of heterogeneity

Possible sources of heterogeneity between organisations were reporting sources, seasonality (winter (November–April) and summer (May–October)) and the size of the outbreak. We investigated these through descriptive analyses, graphical analyses and where possible through fitting Poisson log-linear models.

## Results

**Fig. 1.**
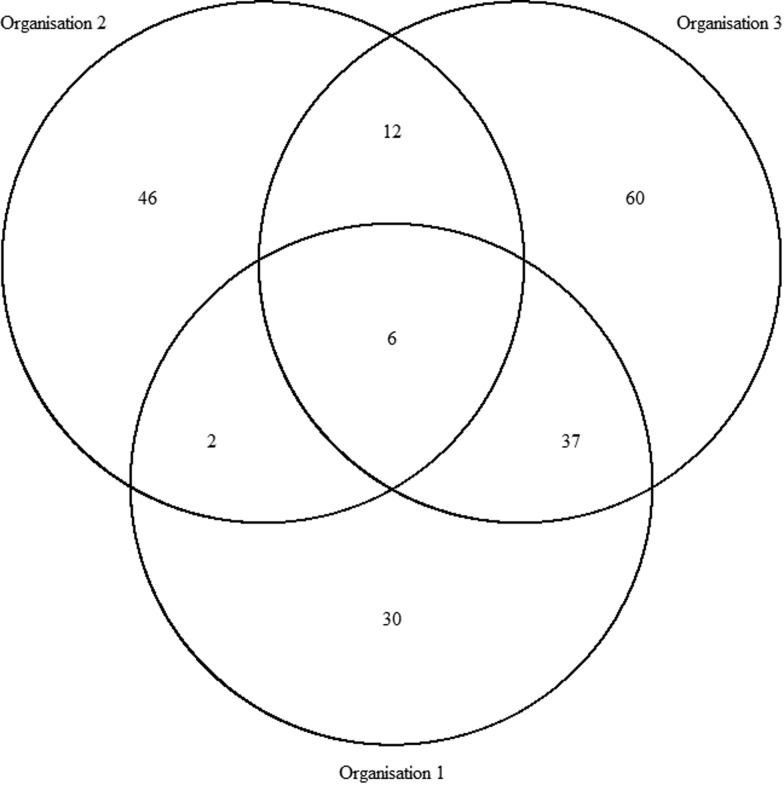
Co-occurrence of outbreaks within the three sources.

In total, there were 193 distinct outbreaks which were ascertained in at least one of the lists. Of these, 149 outbreaks occurred in winter and 43 outbreaks occurred in summer. O1's list contained 75 outbreaks, O2's list contained 66 outbreaks and O3's list contained 115 outbreaks. The co-occurrence of outbreaks on the different combinations of lists is shown in Figure 1. Overlap was greatest for O1 and O3 and least for O1 and O2. Only six outbreaks were common to all three organisations, whereas 136 outbreaks were only found on one list.

The numbers of outbreaks estimated using the methods of Petersen, Chapman and Chao, Tsay *et al*. are shown in Table 1. Taking account of dependence between the lists the sample coverage-based approach suggested that as the estimated sample coverage was low 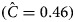 the estimator 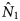 is preferable. The number of distinct outbreaks using the three lists was estimated as 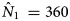 (95% CI 299–457). All estimates were higher than for outbreaks recorded by individual organisations and the number of distinct outbreaks. The only exception to this was the Petersen and Chapman estimates using only the lists from O1 and O3, both of whose confidence intervals overlapped with the number of distinct outbreaks overall (*n* = 193). They did not, however, overlap with the number of distinct outbreaks that the lists for only O1 and O3 had in common (*n* = 127), which were lower than for all other combinations of lists and methods used.

**Table 1.** The estimates of the number of outbreaks of norovirus due to seafood in England, 2004–2011

List combinations	Peterson estimates	Chapman estimates	Chao estimates
Organisations 1 and 2	619 (350–992)	565 (350–992)	NA
Organisations 1 and 3	201 (177–239)	199 (177–239)	NA
Organisations 2 and 3	422 (304–590)	408 (304–590)	NA
Organisations 1, 2 and 3	NA	NA	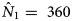 (299–457)

The associations between organisations were assessed using the coefficient of variation. The association between the O1 and O3 lists was positive (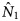 as 0.80), whereas the associations between the O1 and O2 lists was negative (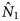 as −0.42) and the O2 and O3 lists were negative (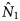 as −0.15). The coefficient of variation helps to explain the discrepancies in the various estimates presented in Table 1. If only lists one and three had been used to derive an estimate of the true number of outbreaks, the positive dependence between the lists would result in the best estimate of outbreaks being underestimated. Conversely, if list two had been used in combination with either list one or list three then the true number of outbreaks would be over-estimated as a consequence of the negative dependence between lists two and one and lists two and three.

The interactions (co-occurrence of outbreaks on different lists) between lists for the whole dataset and for the Winter sub-dataset were modelled using Poisson log-linear models. AICs for all possible combinations of interactions between lists are displayed in Table 2, for full information about the model see Table S1. There were two different models that could not be fitted represented as NA in Table 2. The model containing all possible interactions had as many parameters as data points resulting in it being overfitted. When there was very small overlap between lists 1 and 3 only and lists 2 and 3 only there was numerical instability in model fitting. The most satisfactory model as judged by the AIC allowed for main effects plus an interaction between lists one and three (AIC = 46.1). The main effects represent data for outbreaks appearing on list one only, list two only and list three only. The interactions beyond this for lists one and two only and lists two and three only are small (*n* = 2 and *n* = 12 respectively), whereas the interaction between lists one and three are much larger (*n* = 37). Therefore the effect of assuming independence only assumes independence between list two and the other lists.

**Table 2.** AICs for all interaction models

Interactions	All data - AIC	Abundance (95% profile log-likelihood confidence interval)	Winter data only - AIC	Abundance (95% profile log-likelihood confidence interval)
Including all interactions listed below	NA	NA	NA	NA
Organisations (1,2) + (1,3)	46.1	416 (305–656)	44.8	257 (194–411)
Organisations (1,2) + (2,3)	54.1	239 (216–277)	51.1	174 (159–200)
Organisations (1,3) + (2,3)	NA	NA	NA	NA
Organisations (1,3)	45.2	480 (356–706)	44.6	314 (234–472)
Organisations (1,2)	63.0	276 (243–325)	54.8	188 (168–218)
Organisations (2,3)	62.6	269 (235–322)	62.6	194 (171–230)

The best estimate of the number of outbreaks from this model is 480 (95% profile log-likelihood-based CI 356–706), which is two and a half times higher than that from the distinct number of outbreaks and those reported by individual organisations (*n* = 193). The range of estimated outbreaks using this method overlapped with those from all other estimates with the exception of both the Petersen and Chapman estimates using O1 and O3. The need for information from O2 to be included is borne out by both the Chao estimate and the Poisson log-linear model that indicated that the only important interaction was between lists one and three. There is less overlap between O2 and the other two organisations indicating that it contains extra information.

### Sources of heterogeneity

#### Setting of the outbreak

**Fig. 2.**
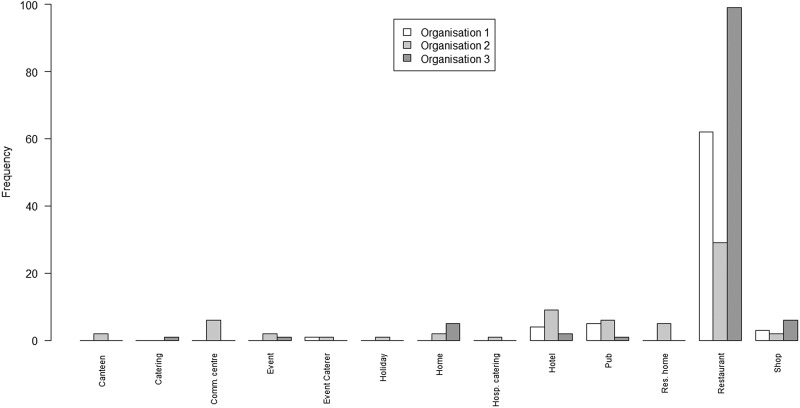
Bar chart of the number of outbreaks by setting and by the organisation

Exploring heterogeneity by setting (where the outbreak occurred), all outbreaks on all lists had a recorded setting. A bar chart of the number of outbreaks from each of a set of settings on each of the three lists is shown in Figure 2.

The principal points of note are that the scope of settings for the lists from O1 and O3 are very similar, with the predominant focus being on restaurants; whereas the scope of settings for O2 is rather wider, covering a range of environments in which food may be served.

### Seasonality

**Fig. 3.**
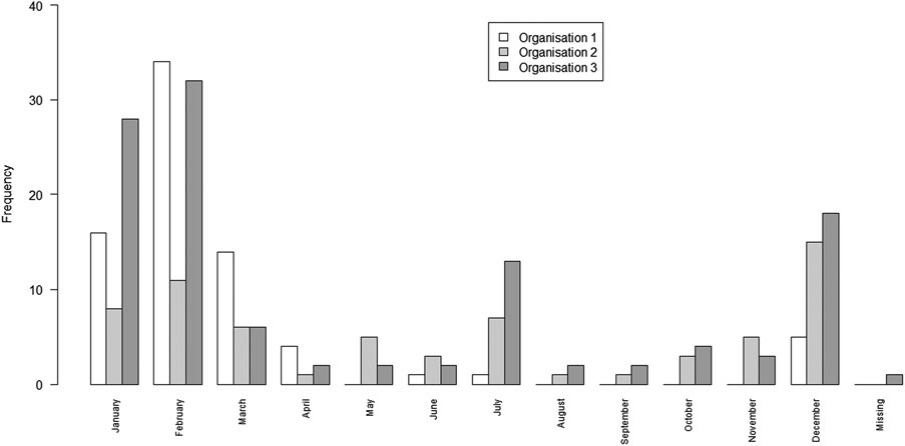
Bar chart of the number of outbreaks by month and organisation, 2004–2011.

The date on which the outbreak began was recorded for 192 of the 193 outbreaks. The pattern of recorded outbreaks by month is shown in Figure 3.

The majority of outbreaks reported by all three organisations occurred in the winter months (*n* = 149) compared with summer months (*n* = 43) (Table 3). The spread of outbreak reporting in O2 and O3 extends across the whole year. In contrast, O1 records no outbreaks from August to November (Table 3). This may be explained by reporting source when comparing the remit of O2 with the differing remits of O1 and O3. The July spike observed for O3 is largely explained by a series of temporally proximate but geographically distinct outbreaks in 2007, which relate to a common risk factor. The July outbreaks for O2 differ entirely from those for O3 and are spaced throughout the study period (one in 2004; three in 2005; two in 2009; and one in 2011). One outbreak was excluded from this subgroup analysis because its season of occurrence was unrecorded. Poisson log-linear models were carried out for winter data and included all possible combinations of interactions between lists. AICs from all fitted models are again displayed in Table 2.

**Table 3.** Frequency of occurrence of outbreaks on different combinations of organisational lists throughout the year, and divided into Summer and Winter

List combination	All data N (%)	Summer N (%)	Winter N (%)
Organisation 1, 2 and 3	6 (0.04)	0 (0.00)	6 (0.04)
Organisation 1 and 2	2 (0.01)	0 (0.00)	2 (0.01)
Organisation 1 and 3	37 (0.24)	1 (0.02)	36 (0.26)
Organisation 2 and 3	12 (0.06)	3 (0.07)	9 (0.07)
Organisation 1 only	30 (0.19)	1 (0.02)	29 (0.18)
Organisation 2 only	46 (0.19)	17 (0.40)	29 (0.20)
Organisation 3 only	59 (0.26)	21 (0.49)	38 (0.24)

Considering the winter models displayed in column three of Table 2, as in the whole dataset model inclusion of the interaction between lists one and three only produces the simplest model with the lowest AIC (AIC = 44.6), suggesting that the interaction between the lists of O1 and O3 is important for the estimation of the number of Winter outbreaks. The corresponding estimate of the number of winter outbreaks is 319 (95% profile log-likelihood-based CI 233–471). This is substantially higher than the distinct number of outbreaks and the number of outbreaks recorded by each organisation.

#### Source of reporting

The recording of the reporting source is very comprehensive for all three organisations (95%, *n* = 71 for organisation 1; 100%, *n* = 66 for O2; 87%, *n* = 100 for O3). O1 and O3 receive the majority (89%, *n* = 63 and 78%, *n* = 78) of their outbreak reports from local government-based sources; in contrast, O2 receives the majority (59.1%, *n* = 39) of its outbreak reports from health-related sources e.g. hospitals. On occasion, outbreaks which have been reported by one organisation to another do not feature on the original organisation's list.

#### Size of outbreak

Of the 193 outbreaks, 98.4% (*N* = 190) reported at least one estimate of the number of people affected. For outbreaks reported to more than one organisation the number of people affected, where recorded, varied between organisations. This may be explained by time-point during the outbreak i.e. at the start, middle or end, that the organisation was contacted. Just one outbreak indicated more than 500 people affected and for seven distinct outbreaks, the number affected indicated 50 or more cases. The majority of outbreaks for which a count was recorded indicated between 1 and 49 cases in total for 182 outbreaks. All but one of the larger outbreaks were recorded by more than one organisation.

## Discussion

This is the first time that an estimate of the burden of outbreaks associated with seafood in England has been made. The combined estimates were more than three times as high the individual count from O3, which captures more outbreaks than the other two organisations. The higher estimates from the combined approach suggest that substantial under-reporting of NoV outbreaks takes place. The findings are reliant on the accuracy of the matching of outbreaks between the three different sources. Care was taken to ensure this through the simultaneous consideration of outbreak location and date of occurrence where provided, with clear criteria concerning what constitutes the same outbreak being specified from the outset.

The consequences of under-reporting are many-fold. First, under-reporting may affect the way that food producers, food handlers and the general public handle, prepare and consume seafood, since their perceived risk of food poisoning may be lower than the actual risk. Second, secondary (and other subsequent) transmission will be underestimated following the initial underestimating of cases infected from contaminated seafood [24] assuming that the numbers of secondary cases are low or have not contacted health services. Finally, without taking under-reporting properly into account the public health significance of NoV may be greatly under-estimated, with implications for infection and disease control.

Capture-recapture methods provide a practical and statistically robust way of accounting for under-reporting of gastrointestinal disease outbreaks caused by non-notifiable agents such as NoV that is only notifiable if foodborne. The use of multiple lists together with explicit modelling of the correlation between lists allows for a more accurate estimation of both the number of outbreaks and associated uncertainty. The difference in the number of estimated outbreaks for different combinations of lists reflects the level of dependence between the lists, with negative dependence between organisations yielding a higher outbreak estimate and a high positive dependence a similar estimate to the numbers of outbreaks on the lists. When estimating the number of outbreaks of a non-notifiable organism, we suggest that where possible a minimum of three sources of information should be taken into account, since with fewer than three lists the modelling of dependence between sources is impossible and the inclusion of such dependence is important for providing an accurate representation of the under-reporting process.

Poisson log-linear models could only be fitted to winter outbreak data and not summer data due to data sparsity. O1 received no reports in summer. There are several reasons why reported summer NoV levels may be low and these may be separated into issues related to contamination and food consumption, and issues related to the reporting process. Considering the former, levels of norovirus in the UK produced oysters are shown to be higher during winter than summer [25, 26]. The effects of this could be exacerbated by specific consumption patterns, for example, consumption of shellfish on St. Valentine's Day [27]. In contrast, traditionally, people were deterred from consuming of seafood in the summer in the UK (‘not eating shellfish when there was no r in the month’), primarily due to the reproductive cycle of certain shellfish, for example the native oyster (*Ostrea edulis*), leading to poor eating condition [28] and this may lead to lower consumption of shellfish in the summer months in the UK. However, the majority of UK oyster growers farm Pacific oysters (*Crassostrea gigas*) that can be eaten throughout the year so shellfish are available all year around. Considering the possible effects of the reporting process, summer cases of gastrointestinal illness may be less likely to be reported as suspected NoV due to the perception that NoV is predominantly a winter disease, and this will have an obvious consequence for the reported number of cases reaching national statistics.

This study could not be replicated for other food sources such as lettuce and raspberries [29, 30] which have been contaminated with NoV and implicated in outbreaks. The pathway by which foods other than seafood are tested for NoV in England is very different from the seafood testing pathway and this limits the applicability of capture-recapture approaches in such contexts.

Dependence between organisations has previously been taken into account using three lists or more in epidemiological capture-recapture studies, for example, Dunbar *et al*. estimating tuberculosis cases [31]. This may be explained through the mechanisms by which the organisations receive their reports. The use of the capture-recapture methodology in the context of estimating the burden of NoV associated with seafood is novel. The outbreak counts held by the different organisations depend upon the mechanisms by which outbreak investigations and illnesses are reported. For example, in a study of *Salmonella* outbreaks in France, reporting to one organisation was more likely if an outbreak was community-based, and reporting to a different organisation was more likely if the outbreak was caused by a particular strain of *Salmonella*. In contrast, positive dependence was likely between the two organisations that were associated with food [11], the same factor that led to overlap between two of the organisations in this study (one and three). This phenomenon commonly occurs when observing interactions between diagnostic surveillance centres and centres which collect incidences of notifiable illnesses, for example, as found by Nardone *et al*. [32] when evaluating the surveillance of Legionnaires’ disease in France. Capture-recapture approaches are useful for integrating surveillance systems whose dependencies vary due to data collection [12] and interpreting the sensitivity of mandatory reporting and associated attributable factors [32].

This is the first time that the burden of NoV outbreaks due to seafood consumption in England has been estimated using capture-recapture on datasets from three different agencies. This study has shown that the burden of outbreaks associated with shellfish is much more substantial than the partial view afforded by interrogating surveillance data held by individual agencies. Using data from different agencies could take into account reporting differences due to the remits of the agencies involved and utilise available data more effectively. What is required is that the three agencies involved co-operate to produce an integrated surveillance system that will improve estimates of the burden of NoV associated with shellfish and provide better intelligence for public health action.

## Supplementary material

For supplementary material accompanying this paper visit https://doi.org/10.1017/S0950268818003217. click here to view supplementary material
