# Supplementary material for: The use of capture-recapture methods to provide better estimates of the burden of norovirus outbreaks from seafood in England, 2004–2011
Source: Epidemiol Infect. 2018 Dec 4;147:e65. doi: 10.1017/S0950268818003217 (PMC6518598; doi:10.1017/S0950268818003217)
Supplement: Supplementary file 1 [file S0950268818003217sup001.docx]

**Epidemiology and Infection**

**THE USE OF CAPTURE-RECAPTURE METHODS TO PROVIDE BETTER ESTIMATES OF THE TRUE BURDEN OF NOROVIRUS OUTBREAKS FROM SEAFOOD IN ENGLAND, 2004-2011.**

**Hardstaff^1*^ J.L., Clough^1^ H.E.,,Harris^1,2^J.P., Lowther^3^ J.A., Lees^3^ D.N. and O’Brien^1,2^ S.J.**

**Supplementary material**

**Supplementary Table S1. The poisson models showing main effects, interactions and fit of the model.**

| Model | Intercept | Main effect org 1 | Main effect org 2 | Main effect org 3 | Interaction org 1, org 2 | Interaction org 1, org 3 | Interaction org 2, org 3 | Residual deviance | AIC |
| --- | --- | --- | --- | --- | --- | --- | --- | --- | --- |
| [1,2] only | 4.444 | -0.773 | -0.917 | -0.349 | -1.208 | - | - | 20.09 | 63.01 |
| [1,3] only | 5.677 | -2.358 | -1.848 | -1.547 | - | 1.842 | - | 2.33 | 45.25 |
| [2,3] only | 4.365 | -0.964 | -0.817 | -0.114 | - | - | -0.867 | 23.57 | 66.49 |
| [1,2] and [1,3] | 5.438 | -0.285 | -1.609 | -1.347 | -0.516 | 1.639 | - | 1.2 | 46.12 |
| [1,2] and [2,3] | 3.885 | -0.483 | -0.143 | 0.209 | -1.498 | - | -1.19 | 9.21 | 54.12 |
| [1,3] and [2, 3] | - | - | - | - | - | - | - | - | - |
